# Science – A life fully lived: Joe Sodroski wins the 2006 Retrovirology Prize

**DOI:** 10.1186/1742-4690-3-45

**Published:** 2006-07-27

**Authors:** Andrew ML Lever

**Affiliations:** 1 Department of Medicine, University of Cambridge, Level 5, Addenbrooke's Hospital, Hills Road, Cambridge, CB2 2QQ, UK

## Abstract

The 2006 M Jeang Retrovirology Prize for HIV research has been awarded to Dr Joe Sodroski

## Interview

**Figure 1:**
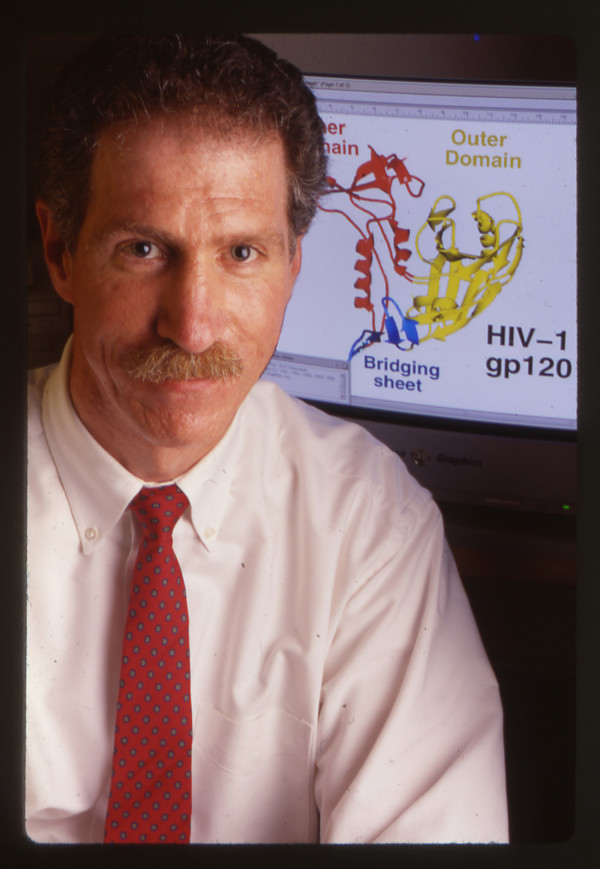
Dr Joe Sodroski winner of this year's Ming K Jeang award.

In 2005 thanks to the generosity of Ming K. Jeang Foundation, an educational foundation based in Houston, Texas, the M Jeang Retrovirology prize was inaugurated [1]. The award goes to a scientist in mid career who in the opinion of the panel of judges [2] has, from the list of nominations, made the most significant contribution to the field. Awards for HIV related and non HIV related research alternate yearly. Last year's winner was Stephen Goff [3]. This year's winner of the Retrovirology Prize for work in the HIV field has been won by Joseph Sodroski (Fig 1).

Dr. Sodroski is Professor of Pathology at the Dana-Farber Cancer Institute, Harvard Medical School and Professor of Immunology and Infectious Diseases at Harvard School of Public Health. Working in the laboratory of Dr. William Haseltine, initially with Dr. Craig Rosen, Dr. Sodroski first demonstrated that HTLV-1 and HIV encoded transactivating proteins Tax and Tat, respectively. Dr. Sodroski also identified the Rev gene, which controls the switch from early to late stages in the replication cycle of HIV. He then studied the molecular and structural biology and pathological effects of the HIV envelope glycoproteins. This culminated in the first X-ray crystal structure of the external glycoprotein, gp120, work done with Drs. Peter Kwong, Richard Wyatt and Wayne Hendrickson. Dr. Sodroski developed the simian-human immunodeficiency virus (SHIV) model in monkeys, in collaboration with Dr. Norman Letvin, and created the first HIV-based vectors. Most recently his group identified Trim5alpha as the restriction factor mediating post-entry blocks to HIV in Old World monkeys. I took the opportunity to ask Dr. Sodroski a wide-ranging set of questions about science, HIV and research in general.

AMLL. Science is obviously a field in which you feel comfortable and have succeeded yet your first degree is an MD. When you originally chose Medicine would you have predicted that this was the route that you would have followed or was there something that drew you away from clinical work into research?

JS. My first love was science, but I was drawn to medicine and the life sciences because of their complexity and obvious benefit to humanity. Medical school instilled in me a life-long appreciation of the pathogenesis and treatment of human disease. Ultimately, I realized that there were only twenty-four hours in a day and decided to pursue what I loved most.

AMLL. HIV was barely known about during your early training. What areas of research, if any, were you considering at that time?

JS. I began studying retroviruses because they were involved in the activation of cellular oncogenes. My first research in Bill Haseltine's lab studied the functional differences between the viral v-fes oncogene and the cellular proto-oncogene, c-fps/fes. Soon thereafter, it became clear that human T-cell leukemia viruses (HTLVs) were inducing leukemias/lymphomas in humans by a mechanism that fundamentally differed from those employed by either leukemia or sarcoma viruses in animals. Our studies revealed that HTLV encodes a transactivator protein, Tax, that accounts for T-cell transformation by this virus. When human immunodeficiency virus (HIV-1) was isolated, we discovered an even more complex repertoire of regulatory proteins. Over the years, the clinical importance of HIV-1 justified giving it an increasing share of my attention.

AMLL. The beginning of your research career coincided with the emergence of pathogenic human retroviruses HTLV-1 and HIV so was it a case of being in the right place at the right time or would you have deliberately moved towards the retroviral area of research?

JS. Being in the right place at the right time was certainly part of the story. Human retroviruses had just been discovered, and Bill Haseltine's friendship with Bob Gallo allowed us early access to key reagents. The other important part of the story, however, was our mental preparedness to discover novel aspects of these retroviruses and how they induce disease. Chance favors only the prepared mind, as Louis Pasteur phrased it.

AMLL. You worked closely with Bill Haseltine and a number of other very well known scientific characters in the early days of HIV research. What lessons did you learn from them?

JS. Bill Haseltine recognized the impact that HIV-1 would have on global health long before the devastating nature of the AIDS pandemic became apparent. So in those early days, Bill and Bob Gallo and their colleagues, myself included, shared a real sense of making an impact on the history of the world with our work. In addition, Bill taught me the power of rigorous thinking to reveal answers and clarify murky research areas.

AMLL. You clearly have enormous enthusiasm for your work. What is it about science that keeps you engaged and what would you say to people considering a scientific career are the biggest attractions and also the biggest problems to be prepared for?

JS. A scientific life is fully lived, with its share of agony and ecstasy. On occasion, scientists can enjoy the special privilege of learning something that no one else knows. Sharing these experiences with the many dedicated and like-minded colleagues that we train and collaborate with is enormously fulfilling. In return for these privileges, scientists live with the constant need to prove themselves capable of making the next novel discovery. Some of this demand is self-imposed, but also derives from the requirement to attain funding.

AMLL. Scientific funding is always under fire for being inadequate. Do you think this reflects a lack of appreciation on the part of funders or simple pragmatism? How would you persuade the powers that be that more would be better?

JS. The American public and Congress generally are supportive of medical research. Unfortunately, federal expenditures in response to other crises have recently limited the financial resources of the National Institutes of Health. The impact of funding limitations will be slower progress; moreover, some individuals that might otherwise choose a scientific career will select other options. This is unfortunate when the availability of so much novel information about genomes and proteomes presents many opportunities for advancing the way medicine is practiced.

AMLL. How do you feel that the research world has changed since you began your work and is it for the better?

JS. Large-scale, empirical approaches to scientific discovery have arrived and are well-suited to particular kinds of research problems. There remains a need for hypothesis-driven science from individuals as well. One of our future challenges is educating sufficient numbers of high-quality scientists suited to these different niches.

AMLL. If you had to guess how the HIV epidemic will evolve globally what would your predictions be?

JS. The course of HIV-1 will be dictated by prevalence, transmission rates, and death rates. HIV-1 prevalence has probably achieved equilibrium in some countries and, in other places where the virus has been introduced more recently, prevalence will likely rise. I expect that, even in the most optimistic scenario, HIV-1 infection and its sequelae will remain a major health problem in the foreseeable future.

AMLL. Given that (as yet) there is no documented case of anyone becoming infected with HIV and subsequently clearing the virus how do you view the huge investment in vaccine approaches to prevention of infection?

JS. Prevention of new HIV-1 infections is the key to changing the course of HIV-1 in the world. Traditionally, vaccines have been successful in preventing the transmission of other viruses. Making a vaccine for a persistent virus like HIV-1, however, involves many challenges. We don't yet have a clear idea of what a successful HIV-1 vaccine will look like. We'll need the concerted efforts of many individuals and should be open to creative, non-traditional approaches to blocking HIV-1 transmission. Hopefully, novel ideas that translate into practical solutions will emerge from the many collaborative groups that have been organized and recently funded.

AMLL. As one of the earliest demonstrators of the practicality of HIV and its family as gene vectors where do you think this area of research will go and will it develop into mainstream medicine?

JS. It's very likely that gene therapy will become the standard therapy for some conditions in the future. So far, lentivirus vectors have been useful as research tools. We'll need to wait to see which gene delivery vectors emerge as preferred clinical modalities.

AMLL. A mid-career prize implies that the next stage is late-career. Will you be happy to continue (funding permitting) as a research scientist through to retiring?

JS. I'm very happy in my present situation and would be quite satisfied if I could continue to contribute to scientific research until retirement.

AMLL. There is a famous piece of advice which is 'Never take advice' but do you have any thoughts which you would pass on to those starting in science which you would have appreciated being told when you began?

JS. 1. Identify and pursue important research problems.

2. Trust your reason and intuition.

3. Persist.

4. Be objective.

5. Appreciate (not necessarily in this order) your students, fellows, collaborators, spouse and family.
